# Drying Process of Waterborne Paint Film on Bamboo Laminated Lumber for Furniture

**DOI:** 10.3390/polym15051288

**Published:** 2023-03-03

**Authors:** Jie Chen, Ying Zhao, Shaofei Yuan, Jian Zhang, Qin Li, Hongyan Wang

**Affiliations:** Key Laboratory of Bamboo Research of Zhejiang Province, Zhejiang Academy of Forestry, Hangzhou 310023, China

**Keywords:** waterborne paint, drying technology, film properties, response surface

## Abstract

In this study, bamboo laminated lumber for furniture was coated with waterborne acrylic paints. The effects of different environmental conditions (including temperature, humidity and wind speed) on the drying rate and performance of the waterborne paint film were investigated. Then, the drying process was optimized using the response surface methodology, and the curve model of drying rate was established, which can provide a theoretical basis for the drying process of the waterborne paint film for furniture. The results showed that the drying rate of the paint film changed with the drying condition. With an increase in temperature, the drying rate increased, and the surface and solid drying time of the film decreased. Meanwhile, with an increase in humidity, the drying rate decreased and the surface and solid drying time increased. Moreover, the wind speed can influence the drying rate, but the wind speed does not significantly affect the surface and solid drying time. The adhesion and hardness of the paint film were unaffected by the environmental conditions, but the wear resistance of the paint film was affected by the environmental conditions. Based on the response surface optimisation, the fastest drying rate was realised at a temperature of 55 °C, humidity of 25% and wind speed of 1 m/s, and the optimal wear resistance was realised at a temperature of 47 °C, humidity of 38% and wind speed of 1 m/s. The paint film drying rate reached the maximum value in 2 min and tended to remain constant after the film was completely dried.

## 1. Introduction

Recently, waterborne paints (WBPs) have been widely used for furniture, decoration and automotive, among others [1]. With the continuous development of the WBPs industry, the manufacturing of bamboo products has adopted a new direction; the application of WBPs to bamboo furniture has gradually become mainstream [2]. WBPs have lower volatile organic compound emissions than traditional solvent-based paints and the advantages of being non-toxic, safe and eco-friendly, which are in accordance with the current production requirements [3]. The research on WBPs has become a hotspot. Several studies in particular have been conducted to modify WBPs, focusing on cross-linking modification, composite modification and nano-modification [4,5,6,7,8,9,10,11,12,13]. In addition, the effect of the finishing process of WBPs on film performance has been studied [10]. However, owing to the large specific heat capacity of water, the energy required for thermal motion in the practical application of WBPs is greater than that of traditional solvent-based paints. This results in a slow film-forming speed that can be easily influenced by temperature, humidity and wind speed [14,15]. Moreover, the performance of WBP films changes with a change in drying temperature and humidity; excessive drying temperature can cause orange peels and pinholes in WBP films. Thus, the drying process of WBPs is a bottleneck that restricts its promotion and development. At present, the research on the drying process of WBPs is primarily focussed WBP films on wood and hardly on the WBPs on bamboo products [16,17,18]. In this study, the bamboo laminated lumber for furniture was coated with waterborne acrylic paints. Moreover, the effects of different environmental conditions (including temperature, humidity and wind speed) on the drying rate and performance of the WBP film were investigated herein. Then, the optimum drying process was selected, and a paint drying model was established, which could provide a theoretical basis for the drying process of the WBPs for furniture.

## 2. Materials and Methods

### 2.1. Materials

Bamboo laminated lumber, made of side-pressured carbonised bamboo sheets, was cut to 100 × 100 × 5 mm^3^ in size. The surface of the bamboo laminated lumber was not coated, and the moisture content was maintained below 10%.

Waterborne acrylic paints, which were bought from enterprises, were divided into primer (D) and topcoat (M). The main performance parameters of the paints are listed in Table 1.

Other materials used in this study are as follows: strips of gauze, absorbent cotton, a high-grade drawing pencil from a Chinese brand, qualitative filter paper and scotch tape.

### 2.2. Major Equipment

The major equipment used in this study included a paint film drying time tester, a pencil hardness tester, a paint film scriber, a temperature humidity chamber, a paint film wear-resistance tester and more.

### 2.3. Preparation of Paint Films

**Figure 1:**
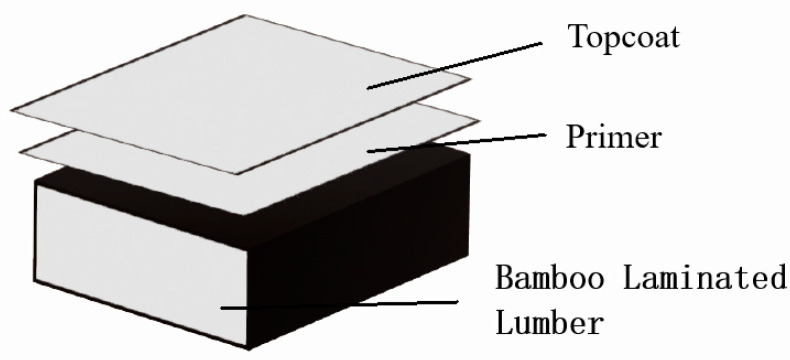
Painting process.

In this study, bamboo laminated lumber was finished with one-layer primer coating and one-layer topcoat coating (Figure 1). Moreover, the coating weight of primer and topcoat was maintained at 80 ± 5 g/m^2^. Before the primer coating, the bamboo laminated lumber was placed at room temperature and sanded with a 240 # sandpaper until the surface became smooth. Before the topcoat coating, it was also sanded with 320 # sandpaper until the surface was smooth. After finishing, it was immediately dried in a temperature and humidity chamber. The single-factor experimental method was used; Table 2 lists the drying conditions of the WBP film used in this study.

Based on a single-factor experiment, the experimental range with high-performance parameters was selected for response surface optimisation, and the parameters were optimised. Under the optimal drying condition, the drying rate of the paint film was tested, and its curve model was established.

### 2.4. Testing and Characterisation

The drying rate and hardness of the paint film were tested according to the GB/T 1728–2020 and GB/T 6739–2006 standards, respectively, whereas the wear resistance and adhesion of the paint film were tested according to the GB/T 4893.8–2013 standard. Each indicator was tested five times, and the average of the results was calculated. Before the performance tests, balancing the completely dried paint film in the natural environment for one week was necessary; each test was performed at room temperature. All experiments were repeated five times, and the experimental error was within 10.0%.

## 3. Results and Discussion

### 3.1. Effect of the Drying Process on the Drying Rate of Paint Films

#### 3.1.1. Effect of Drying Temperature on the Drying Rate of Paint Films

**Figure 2:**
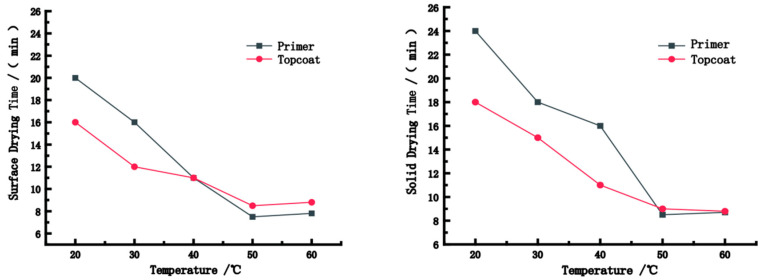
The surface and solid drying time of the paint films at different temperatures.

Figure 2 shows the surface and solid drying time of the paint films at different temperatures. Under the same wind speed and humidity conditions, with an increase in temperature, the surface and solid drying time decreased, and the drying rate increased. This may be because the energy required for the evaporation of water molecules in the paint film increases with the temperature; hence, the speed at which water molecules move from inside the coating to the surface increases [19,20]. The longest surface/solid drying time of the paint film in this experiment was at a temperature of 20 °C; the surface (solid) drying time of the primer and topcoat were 20 (16) and 20 (18) min, respectively, due to the slow drying rate. The surface (solid) drying time of the primer and topcoat were 7.5 (8.5) and 8.5 (9) min, respectively, when the paint film was dried at 50 °C. The drying rate reached the maximum at about 50 °C because the surface/solid drying time did not decrease when dried at 60 °C. This showed that the drying rate was close to saturation at 50 °C and the movement speed of water molecules tended to be stable. Therefore, the effect of temperature increase on the drying rate decreases gradually when the temperature exceeds 50 °C. From Figure 2, the drying rate of the topcoat was faster than that of the primer because, on the one hand, different additives added resulted in different drying rates, while on the other hand, water penetrated inward along the vessel of the bamboo laminated lumber in the drying process of the primer. Moreover, the particles accumulated between each other, but water molecules only diffused into the air during the drying process of the topcoat. With comprehensive consideration, we recommend 50 °C as the optimum drying temperature.

#### 3.1.2. Effect of Relative Humidity on the Drying Rate of Paint Films

**Figure 3:**
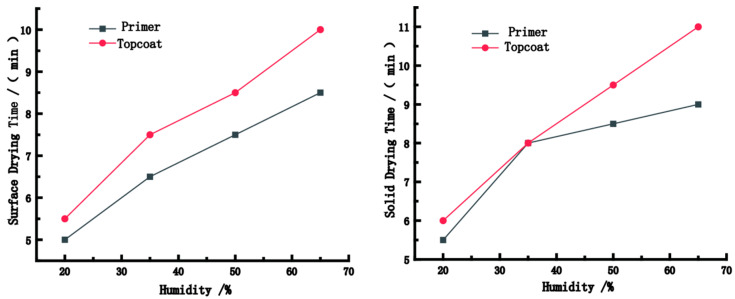
The surface and solid drying time of the paint films under different humidity values.

Figure 3 shows the surface and solid drying time of the paint films under different humidity values. Under the same temperature and wind speed conditions, with the increase in humidity, the surface and solid drying time increased and tended to remain stable. The longest surface/solid drying time of the paint films in this experiment was observed under a humidity of 65%; the surface drying and solid drying time for the primer were 8.5 min and 10 min, respectively, and that for the topcoat were 9 min and 11 min, respectively. The dying rate reached its maximum value under a humidity of 20%, where the surface and solid drying time of the primer were 5 min and 5.5 min, respectively, and that of the topcoat were 5.5 min and 6 min, respectively. The increase in relative humidity led to the increase in the ability of the substrate to absorb water continuously, decreasing the drying rate of the paint film. However, owing to the excessive density of water molecules in the air, the ability of water molecules inside the coating to diffuse into the air was affected. Therefore, with the increase in relative humidity, the drying rate of the paint film decreased. In addition, owing to the loss of heat on the coating surface, the numerous water molecules in the air can liquefy on the coating surface, forming small water droplets that condense on the surface of the paint film, resulting in the phenomenon of a white paint film. The thickness of this white paint film increased with relative humidity. Therefore, to avoid this phenomenon and improve the drying rate, the humidity of 20% is optimum.

#### 3.1.3. Effect of Wind Speed on the Drying Rate of Paint Films

**Figure 4:**
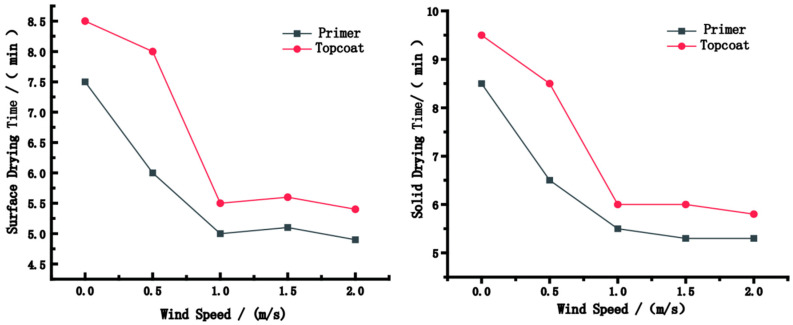
The surface and solid drying time of the paint films under different wind speeds.

Figure 4 shows the surface and solid drying time of the paint films under different wind speeds. The wind speed improved the drying rate of paint films marginally. The increase in wind speed increased the speed of airflow; thus, water molecules that accumulated on the surface of the film dispersed quickly into the air. Hence, the density of water molecules on the surface of the paint film decreased. However, the drying rate did not increase with wind speed; the surface and solid drying time remained unchanged when the wind speed reached 1 m/s.

The longest surface/solid drying time of the paint film in this experiment was observed under a wind speed of 0 m/s; the surface and solid drying time of the primer were 7.5 min and 8.5 min, respectively, and those of the topcoat were 8.5 min and 9.5 min, respectively. The dying rate reached its maximum value under a wind speed of 1 m/s, where the surface drying and solid drying time of the primer were 5 min and 5.5 min, respectively, and those of the topcoat were 5.5 min and 6 min, respectively. Excessive wind speed caused cracks, crusts, wrinkles and other adverse effects on the paint film. Choosing an appropriate wind speed can improve the quality of paint film; thus, a wind speed of 1 m/s is optimum for the drying process of the paint film.

### 3.2. Effect of the Drying Process on the Performance of Paint Films

#### 3.2.1. Hardness and Adhesion of Paint Films

Table 3 shows the performance of adhesion and hardness of WBP films on the bamboo laminated lumber under different drying conditions. Adhesion refers to the degree of mutual adhesion between the coating and the surface of the plate due to the physical or chemical reaction, and hardness refers to the ability of the film to resist deformation [21,22,23,24,25]. Under different temperature, relative humidity and wind speed conditions, the adhesion and hardness of the paint film remained constant at grades 0 and B, respectively; thus, the drying process had no clear effect on the adhesion and hardness of the paint film. This is because the adhesion and hardness of the paint film are determined by the properties of WBPs.

#### 3.2.2. Wear Resistance of the Paint Film

**Figure 5:**
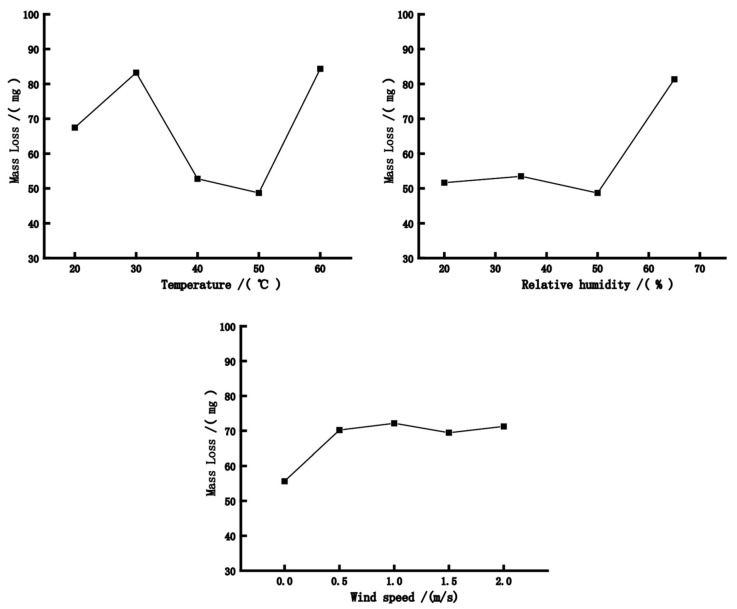
The mass loss of the paint film under different drying conditions.

Wear resistance refers to the ability of the film to resist wear. Figure 5 shows the mass loss of the WBP film on the bamboo laminated lumber under different drying conditions, which reflects the wear resistance. The mass loss first increased and then decreased with temperature. However, when the temperature exceeded 50 °C, the mass loss again increased. This is because the curing speed of the coating is slow and emulsion particles move slowly at low temperatures, resulting in insufficient uniformity of the film; hence, it exhibits poor wear resistance. With the increase in temperature, the movement of emulsion particles became rapid, which improved the uniformity of the paint film leading to the increase in its wear resistance. In this experiment, the paint film structure achieved its best state at 50 °C. When the temperature exceeded 50 °C, the structure was damaged, resulting in decreased wear resistance. For the humidity values below 50%, the wear resistance of paint film remained unaffected; however, the mass loss increased considerably when the humidity values exceeded 50%. This is probably because the increase in relative humidity leads to the increase in the water content of the substrate, which affects the mechanical properties of the paint film and decreases its wear resistance. The mass loss of the paint film increased significantly when drying in the wind, but it had no significant effect on the mass loss of the paint film; the wear resistance tended to stabilise when the wind speed exceeded 0.5 m/s.

### 3.3. Optimisation of the Drying Process Parameters and the Establishment of Drying Curve Using the Response Surface Method

#### 3.3.1. Optimisation of the Drying Process Parameters

The Box–Benhnken experimental design was employed as the response surface method for measuring the solid drying time of the paint film at a temperature of 50 °C, humidity of 25% and wind speed of 1 m/s and for measuring the wear resistance of the paint film at a temperature of 50 °C, humidity of 30% and wind speed of 1 m/s. Table 4 and Table 5 list the results for the solid drying time and wear resistance of the paint film, respectively, using the response surface method.

The multivariate regression equations between drying time (wear resistance) and temperature (A) as well as humidity (B) and wind speed (C) were analysed using Design-Expert 11.0. The multivariate regression equation for drying time and wear resistance are Equation (1) and Equation (2) respectively.

Y = 4.46 − 3.11A − 0.075B − 0.8375C + 0.275AB + 0.3AC − 0.025BC + 3.44A^2^ + 0.37B^2^ + 0.245C^2^ (1)

Y = 48.90 + 15.99A − 5.47B + 2.87C + 4.21AB + 1.98AC − 1.89BC + 17.522A^2^ + 14.21B^2^ + 7.60C^2^ (2)

Table 6, Table 7, Table 8 and Table 9 show the variance results of the quadratic models for solid drying time and wear resistance.

The difference in the regression models for solid drying time and wear resistance was significant because the F-values in these models were 684.45 and 186.56, respectively, and the *p*-values were <0.0001. The *p*-values of the lost fitting terms are 0.0746 and 0.0602, respectively, which are >0.05. Therefore, the differences between the models were not significant, indicating that the equations were reliable. The regression coefficients R^2^ for the two models were 99.89% and 99.58% (>85%), respectively, indicating that the equations had a good fitting degree. The regression equation can be used instead of the real point of the test to describe the relation between the variables and response value. The correction coefficients R^2^_Adj_ were 0.9974 and 0.9905, respectively, indicating that the model can explain 99.74% and 99.05% of the solid drying time and wear resistance, respectively. The data in the table showed that the test design was reliable, i.e., the error was small, and it was suitable for the real situation. Hence, this data can be used to analyse and predict the test results of the drying time of paint films [26,27]. Using the above data, the significance of each regression coefficient was tested, and the degree of influence of each factor on the result was determined by comparing their F-values. In this experiment, the degree of influence of each factor for the solid drying time was as follows: temperature > wind speed > humidity; meanwhile, the degree of influence of each factor for the wear resistance was as follows: temperature > humidity > wind speed.

The response surface plots depict the influence of two independent variables on the dependent variable, where the steeper the slope of the plot, the higher the influence of each variable on the response value. When the contour lines are elliptical, the interaction is distinct; conversely, when the contour lines are circular, there is insignificant interaction between the two.

**Figure 6:**
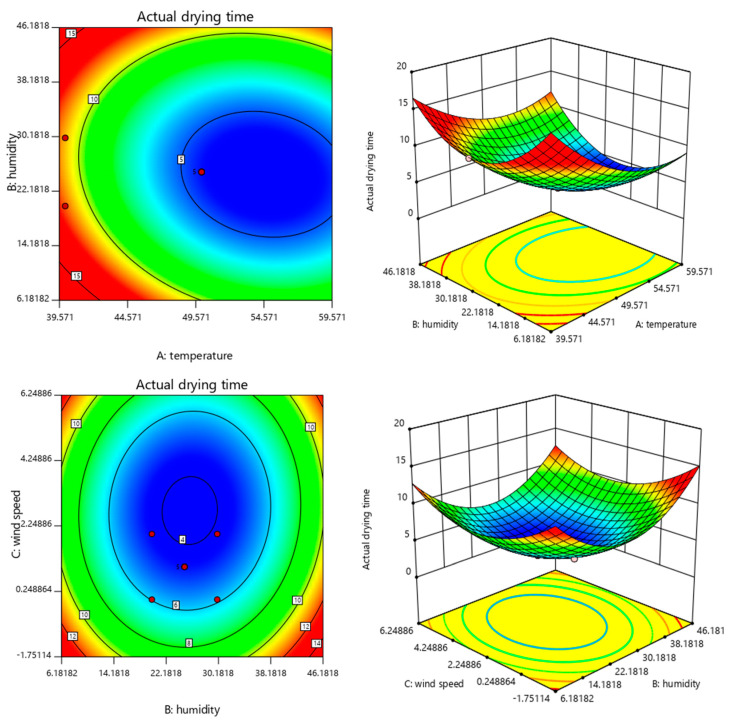

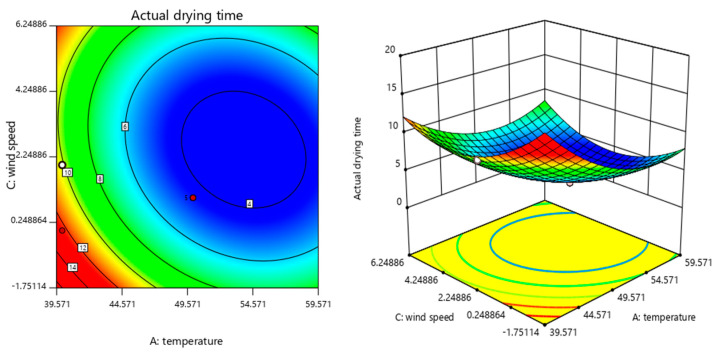
The response surface and contour of temperature, humidity and wind speed for the solid drying time. (Left is the response surface, right is the contour map).

**Figure 7:**
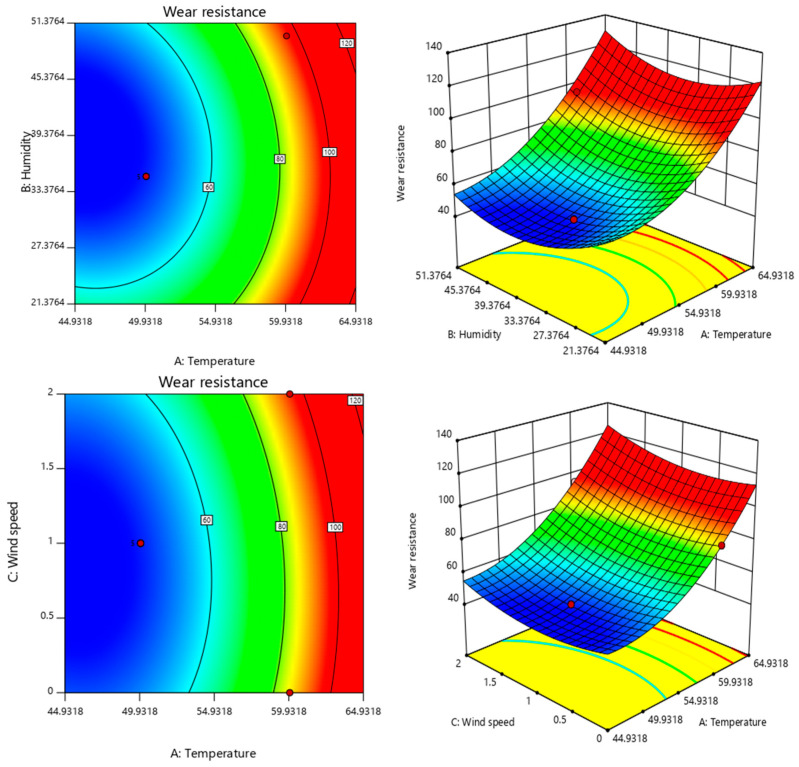

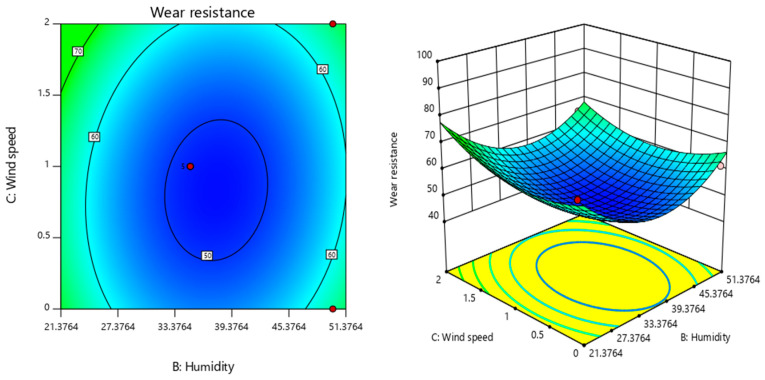
The response surface and contour of temperature, humidity and wind speed for the wear resistance. (Left is the response surface, right is the contour map).

Figure 6 and Figure 7 show the diagrams of the response surface plots as well as the contour of the solid drying time and wear resistance of the paint film, respectively, under the interaction among various environmental conditions, the response surface on the left is on the left and the contour map is on the right. The interaction between temperature and humidity had the greatest effect on the solid drying time, whereas the interaction between humidity and wind speed had the least effect on it. In addition, the interaction between wind speed and humidity has the greatest effect on the wear resistance. Moreover, the wear resistance gradually increases with temperature and wind speed under constant humidity, whereas the wear resistance gradually increases with temperature and humidity under constant wind speed.

Using Design-Expert 11.0 to solve the regression fitting equation for the solid drying time of the paint film, the following optimal environmental condition was derived: a temperature of 56.24 °C, humidity of 24.13% and wind speed of 0.8859-m/s. Under this condition, the predicted solid drying time of the paint film was 3.93011 min. According to the actual operation feasibility, the modified drying curve condition was as follows: a temperature of 55 °C, humidity of 25% and wind speed of 1 m/s. The verification test was performed under this condition and the actual drying time of the paint film was 4.1 min. Similarly, the optimal condition for wear resistance was as follows: a temperature of 46.18 °C, humidity of 37.96% and wind speed of 1.37 m/s. Moreover, the predicted wear resistance was 46.2042 g/m^2^. The modified wear resistance curve condition was as follows: a temperature of 55 °C, humidity of 25% and wind speed of 1 m/s. The verification test was performed under this condition, and the mass loss of the paint film was 48.34 g/m^2^. This was close to the predicted value. The relative errors in the results predicted by the same model were small, indicating that the production process parameters obtained using the response surface method for the process optimisation of the actual drying time and wear resistance of the paint film were accurate, reliable and were practically applicable.

#### 3.3.2. Establishment of the Drying Rate Curve

**Figure 8:**
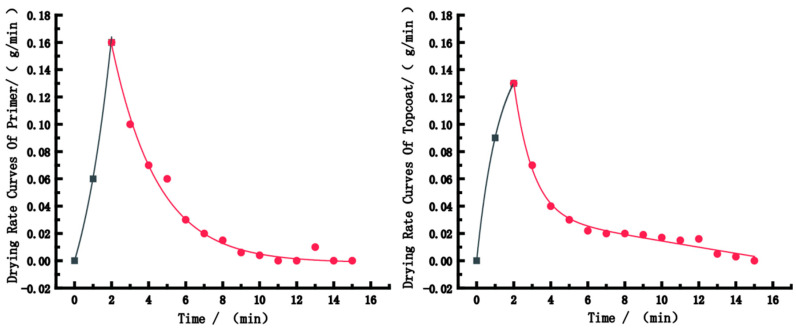
The drying rate curves of the paint films.

Figure 8 shows the drying rate curves of the primer and topcoat. The two curves are similar. The curves first increased, reached their maximum at 2 min, and then decreased, finally tended to 0 m/s. The drying rate of the primer was faster than that of the topcoat, and its maximum value was greater than that of the topcoat. According to the data fitting, the drying rate curve models of the primer and topcoat can be derived as follows. The first and second half of the equation of the drying rate model of the primer are Equations (3) and (4), respectively. The first and second half of the equation of the drying rate model of the topcoat are Equations (5) and (6), respectively.

y = 0.045 × exp(−x/−1.76185) + 0.045 × exp(−x/2.15338) − 0.009(3)

y = 0.12885 × exp(−x/2.47524) + 0.22987 × exp(−x/2.47545) − 0.00139(4)

y = −0.081 × exp(−x/1.10984) − 0.081 × exp(−x/1.35647)(5)

y = 0.68792 × exp(−x/1.02716) + 2906.62 × exp(−x/1.28915) − 2906.5921.(6)

## 4. Conclusions 

In this study, bamboo laminated lumber for furniture was coated with waterborne acrylic paints. The effects of different environmental conditions (including temperature, humidity and wind speed) on the drying rate and performance of the WBP film were investigated. Then, the optimum drying condition was selected, and an empirical model of drying rate was established, which can provide the theoretical basis for the drying technology of WBP films for furniture. The following conclusions were drawn from our analysis.

(1) Temperature, humidity and wind speed influence the drying time and performance of WBP films. The effect of temperature on the surface/solid drying time is more obvious. The adhesion and hardness of WBP films are uninfluenced by environmental conditions, but the wear resistance is affected by environmental conditions.
(2) A response surface optimisation analysis was performed using Design-Expert 11.0 with respect to solid drying time and wear resistance, and the optimal drying condition for the solid drying time is a temperature of 55 °C, humidity of 25% and wind speed of 1 m/s, which yields a solid drying time of 4.1 min. The optimal condition for wear resistance is 47 °C temperature, 38% humidity and 1-m/s wind speed, which yields a 48.34-g/m^2^ mass loss of paint film.
(3) The drying rate curves of the primer and topcoat coatings are established. The two curves are similar; they both first increased, reached their maximum at 2 min and then decreased, finally tending to 0 m/s. The drying rate of the primer was faster than that of the topcoat, and the maximum value was greater than that of the topcoat.

## Figures and Tables

**Table 1:** Main performance parameters of waterborne acrylic paints.

Paints	pH Value	Viscosity/(mPa·s)	Solid Content/%	Particle Size/nm
Primer (D)	7	15.7	34	221.8
Topcoat (M)	7	30.8	38	925.2

**Table 2:** The drying conditions for WBP films.

Paints	Temperature/°C	Relative Humidity/%	wind Speed/m/s
Primer (D)	20, 30, 40, 50, 60	20, 35, 50, 65	0, 0.5, 1, 1.5, 2
Topcoat (M)	20, 30, 40, 50	20, 35, 50, 65	0, 0.5, 1, 1.5, 2

**Table 3:** Adhesion and hardness of the paint film under different environmental conditions.

Temperature/°C	Relative Humidity/%	Wind Speed/m/s	Adhesion	Hardness
20	50	0	0	B
30	50	0	0	B
40	50	0	0	B
50	50	0	0	B
60	50	0	0	B
50	20	0	0	B
50	35	0	0	B
50	65	0	0	B
50	50	0.5	0	B
50	50	1	0	B
50	50	1.5	0	B
50	50	2	0	B

**Table 4:** Results of the solid drying time.

Samples	Temperature /°C	Relative Humidity /%	Wind Speed /m/s	Solid Drying Time /min
1	50	25	1	4.4
2	40	30	1	11
3	50	25	1	4.5
4	60	20	1	5
5	60	25	0	5.7
6	50	25	1	4.6
7	50	30	0	5.8
8	40	25	2	10
9	50	20	0	5.8
10	50	25	1	4.4
11	40	20	1	11.8
12	40	25	0	12.5
13	50	20	2	4.4
14	60	25	2	4.4
15	50	30	2	4.3
16	60	30	1	5.3
17	50	25	1	4.4

**Table 5:** Results of the wear resistance.

Samples	Temperature /°C	Relative Humidity /%	Wind Speed /m/s	Wear Resistance/(g/m²)
1	50	50	0	62.46
2	50	20	2	82.72
3	60	50	1	96.98
4	50	35	1	48.83
5	50	35	1	49.23
6	40	35	2	58.76
7	40	50	1	56.12
8	40	35	0	57.75
9	60	20	1	96.71
10	50	35	1	49.43
11	50	35	1	49.76
12	50	35	1	47.23
13	50	20	0	72.43
14	60	35	2	94.23
15	40	20	1	72.69
16	50	50	2	65.21
17	60	35	0	85.32

**Table 6:** Variance results of the quadratic model for the solid drying time.

Source	Sum of Squares	df	Mean Square	F-Value	*p*-Value
Model	135.96	9	135.96	684.45	<0.0001
A	77.50	1	77.50	3511.38	<0.0001
B	0.0450	1	0.0450	2.04	0.1964
C	5.61	1	5.61	254.23	<0.0001
AB	0.3025	1	0.3025	13.71	0.0076
AC	0.3600	1	0.3600	16.31	0.0049
BC	0.0025	1	0.0025	0.1133	0.7463
A^2^	49.97	1	49.97	2264.04	<0.0001
B^2^	0.5764	1	0.5764	26.12	0.0014
C^2^	0.2527	1	0.2527	11.45	0.0117

**Table 7:** Fit statistics of the solid drying time.

Std. Dev.	0.1486	R^2^	0.9989
**Mean**	6.37	**Adjusted R^2^**	0.9974
**C.V.%**	2.33	**Predicted R^2^**	0.9852
		**Adeq Precision**	72.5138

**Table 8:** Variance results of the quadratic model for the wear resistance.

Source	Sum of Squares	df	Mean Square	F-Value	*p*-Value
Model	5081.95	9	564.66	186.56	<0.0001
A	2045.44	1	2045.44	675.79	<0.0001
B	239.5900	1	239.5900	79.16	<0.0001
C	65.9	1	65.9	21.77	0.0023
AB	70.9	1	70.9	23.42	0.0019
AC	15.6000	1	15.6000	5.15	0.0574
BC	14.21	1	14.21	4.70	0.0669
A^2^	1292.35	1	1292.35	426.98	<0.0001
B^2^	850.15	1	850.15	280.88	<0.0001
C^2^	243.17	1	243.17	80.34	<0.0001

**Table 9:** Fit statistics of the wear resistance.

Std.Dev.	1.74	R^2^	0.9958
**Mean**	67.40	**Adjusted R^2^**	0.9905
**C.V.%**	2.58	**Predicted R^2^**	0.9447
		**Adeq Precision**	36.7087

## Data Availability

Not applicable.
